# Characterization of Drug-Resistant Lipid-Dependent Differentially Detectable *Mycobacterium tuberculosis*

**DOI:** 10.3390/jcm10153249

**Published:** 2021-07-23

**Authors:** Annelies W. Mesman, Seung-Hun Baek, Chuan-Chin Huang, Young-Mi Kim, Sang-Nae Cho, Thomas R. Ioerger, Nadia N. Barreda, Roger Calderon, Christopher M. Sassetti, Megan B. Murray

**Affiliations:** 1 Gemeentelijke Gezondheidsdiensten en Geneeskundige Hulpverleningsorganisaties in de Regio Nederland, 3524 SJ Utrecht, The Netherlands; anneliesmesman@gmail.com; 2 Department of Microbiology and Institute for Immunology and Immunological Diseases, College of Medicine, Yonsei University, Seoul 16995, Korea; backbee2@gmail.com (S.-H.B.); kyoungmi@yonsei.ac.kr (Y.-M.K.); srcho918@yuhs.ac (S.-N.C.); 3 Division of Global Health Equity, Brigham and Women’s Hospital, Boston, MA 02115, USA; abeytw@gmail.com; 4 Department of Computer Science and Engineering, Texas A&M University, College Station, TX 77843, USA; ioerger@cse.tamu.edu; 5 Socios En Salud Sucursal Peru, Lima 15001, Peru; nbarreda_ses@pih.org (N.N.B.); rogercalderon7@gmail.com (R.C.); 6 Department of Microbiology and Physiological Systems, University of Massachusetts Medical School, Worcester, MA 01655, USA; Christopher.Sassetti@umassmed.edu; 7 Department of Global Health and Social Medicine, Harvard Medical School, Boston, MA 02115, USA; 8 Department of Epidemiology, Harvard T.H. Chan School of Public Health, Boston, MA 02115, USA

**Keywords:** diagnosis, tuberculosis, TB, culture, drug-resistant

## Abstract

An estimated 15–20% of patients who are treated for pulmonary tuberculosis (TB) are culture-negative at the time of diagnosis. Recent work has focused on the existence of differentially detectable *Mycobacterium tuberculosis* (*Mtb*) bacilli that do not grow under routine solid culture conditions without the addition of supplementary stimuli. We identified a cohort of TB patients in Lima, Peru, in whom acid-fast bacilli could be detected by sputum smear microscopy, but from whom *Mtb* could not be grown in standard solid culture media. When we attempted to re-grow *Mtb* from the frozen sputum samples of these patients, we found that 10 out of 15 could be grown in a glycerol-poor/lipid-rich medium. These fell into the following two groups: a subset that could be regrown in glycerol after “lipid-resuscitation”, and a group that displayed a heritable glycerol-sensitive phenotype that were unable to grow in the presence of this carbon source. Notably, all of the glycerol-sensitive strains were found to be multidrug resistant. Although whole-genome sequencing of the lipid-resuscitated strains identified 20 unique mutations compared to closely related strains, no single genetic lesion could be associated with this phenotype. In summary, we found that lipid-based media effectively fostered the growth of *Mtb* from a series of sputum smear-positive samples that were not culturable in glycerol-based Lowenstein–Jensen or 7H9 media, which is consistent with *Mtb*’s known preference for non-glycolytic sources during infection. Analysis of the recovered strains demonstrated that both genetic and non-genetic mechanisms contribute to the observed differential capturability, and suggested that this phenotype may be associated with drug resistance.

## 1. Introduction

Tuberculosis (TB) poses a major health problem globally, and clinical care is challenged by the long duration of treatment and the emergence of drug resistance. Microbiological culture of *Mycobacterium tuberculosis* (*Mtb*) is the gold standard test to detect active disease and to diagnose drug resistance by drug-sensitivity testing (DST). However, because mycobacterial culture is slow and must be performed in laboratories that are equipped with advanced biosafety features, clinical diagnosis often depends on sputum smear microscopy. This involves the staining of a smeared sputum sample with a dye (Ziehl–Neelsen) that preferentially detects acid-fast bacilli (AFB); the density of the bacilli observed on the slide is reported on a scale that includes 0, scanty (meaning that fewer than 10 bacilli were observed) and 1–3, with each level indicating an increasing number of bacilli. Compared to culture, sputum smear microscopy is considered to be only about 50% sensitive, with values ranging from 32% to 94% in different studies [1].

Although culture remains the gold standard, many patients who are diagnosed with TB disease have culture-negative (Cx−) sputum samples. Even in settings with good laboratory infrastructure, such as the United States and United Kingdom, over 15% of patients that meet the clinical criteria for a diagnosis of pulmonary TB do not have confirmation by culture [2,3,4]. Cx− TB is often associated with paucibacillary disease, e.g., with early or “incipient” TB or with HIV co-infection [2,5,6]. Therefore, smear-positive/culture-negative TB (Sm+/Cx−) is often thought to reflect the presence of dead bacteria, and these smear results are interpreted as false positives [5]. However, test-related factors, such as medium composition, can affect mycobacterial growth and detection. Several recent reports have documented the presence of *Mtb* subpopulations in clinical samples, or experimental conditions that do not grow in conventional culture without the addition of supplementary stimuli [7,8,9]. These bacteria, variously labelled ‘differentially culturable’ or ‘differentially detectable,’ have a slow-growing, drug-tolerant phenotype, which may be induced by exposure to antibiotics or immune pressure [9,10]. Other recent studies demonstrated that culture in routine mycobacterial growth incubator tube (MGIT) 7H9 or 7H11 media reduces the genetic diversity of mycobacteria that are present in sputum, again suggesting that standard media may select for certain subpopulations and exclude others [11,12].

*Mtb* can use multiple different carbon sources under different growth conditions. During host infection, *Mtb* requires lipids for growth and virulence, and the activation of lipid metabolism genes has been associated with a ‘fat and lazy’ persister-like phenotype [13,14,15,16,17,18,19]. Whereas, lipids are an important fuel source for *Mtb* during infection, the main carbon source in standard culture media is glycerol. We hypothesized that available carbon sources may alter the culturability of *Mtb* in conventional diagnostic media, and that this phenomenon might explain the occurrence of clinical TB samples that are positive by Ziehl–Neelsen staining of the sputum, but negative by routine culture. We investigated this by characterizing the growth characteristics, drug sensitivity, and genomes of ‘culture-negative’ bacilli that were isolated from the stored sputum samples of patients that had been collected in the course of a large cohort study of TB patients in Lima, Peru. 

## 2. Results

### 2.1. Culture-Negative TB in a Peru Cohort Study

Using clinical records and stored sputum samples, we identified patients with Sm+/Cx− TB, who had been enrolled in a prospective cohort study that was conducted in Lima, Peru between 2008 and 2012. In this study, we enrolled 4500 TB patients, in whom we assessed treatment outcomes, and 14,044 of their household contacts [20]. Our study thus included 5192 individuals with TB disease; this included the 4500 who were enrolled when they presented to health centers and the 692 who were detected during the ongoing monitoring of household contacts, which was part of the original study design. Sputum samples were collected at the time of diagnosis and at repeated intervals during the course of treatment; these were assessed by smear microscopy for acid-fast bacilli, culture on Lowenstein–Jensen (LJ) media and by phenotypic drug-sensitivity tests (pDSTs). We also froze and stored decontaminated sputum samples from a subset of patients. 

Among 5192 index patients, 1091 (21%) were Cx− at baseline (Table 1). This high percentage may reflect early detection due to the active follow-up of the 692 household contacts who developed TB. Of these, 304 (28%) patients were Sm+/Cx−. In addition, 760 (15%) of the cases had a sputum sample, obtained during the course of treatment, that was Sm+/Cx−. Thirty-four patients had Sm+/Cx− sputum samples, both at the time of diagnosis and after treatment initiation. Of the 5192 cases, we had pDST results from 3473, of which 462 (13.3%) were diagnosed with multidrug-resistant TB (MDR) and 1179 (33.95%) were resistant to one or more drugs.

### 2.2. Selection of Smear-Positive Culture-Negative Study Samples

**Figure 1:**
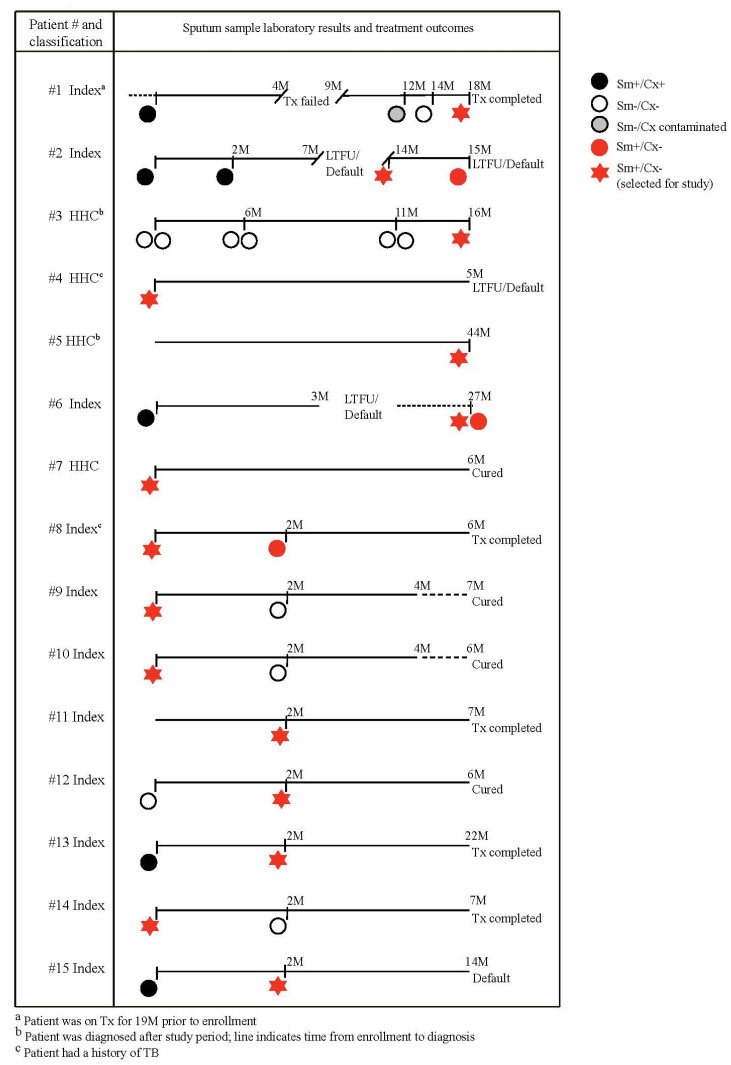
Timelines with sputum sample results and treatment outcomes for the 15 TB patients whose samples were selected.

To investigate the Sm+/Cx− phenotype, we randomly selected 15 of 37 of smear-positive samples for which the smear grade was 3+, but which were Cx−. Eight of these were sputum samples obtained at the time of diagnosis and seven after treatment initiation (Figure 1). Three of the 15 patients had more than one Sm+/Cx− sample. Table 2 provides phenotypic drug-resistance profiles for those samples from these patients that could be cultured on LJ. We also selected 15 baseline samples that were Sm–/Cx− from patients who had either a Sm+ or Cx+ sample during the course of treatment, or received treatment for longer than six months.

### 2.3. Lipid-Dependent Mtb Culture of Sm+/Cx− Sputum Samples

To test the hypothesis that the Cx− phenotype that we had observed might be related to the carbon source in the media, we characterized the growth of *Mtb* from the 30 thawed sputum samples in both glycerol-rich medium and a glycerol-poor/lipid-rich medium. This medium was rich in fatty acids derived from coconut oil, high content, 50% medium chain fatty acids presented as neutral mono-, di-, triglycerides. *Mtb* can readily metabolize fatty acids as a carbon or energy source [15].

Five of the stored Sm+/Cx− sputum samples (#11–15) did not grow in lipid medium (Table 3). The remaining 10 samples (66%) grew in lipid medium, two of which also grew in glycerol-rich medium, although at a slower rate than in the lipid-based medium (Table 3). Three strains (#7–9) only initiated growth following the addition of glycine betaine, which we added to all the cultures after four weeks. The apparent stimulatory effect of this osmoprotectant may indicate that the salts that were introduced during the decontamination process may inhibit the growth of some strains, as described previously [21]. Seven out of ten lipid-grown strains (#1–6, #8) had been collected from patients who had been on treatment for over one year, or who had a history of previous TB treatment (Figure 1; Table 2 and Table 3); three of these 10, all of the patients with phenotypic DST (pDST) baseline results available, had been diagnosed with MDR (#1, #2) or isoniazid (INH) mono-resistance (#6). None of the 15 Sm–/Cx− samples grew in either culture condition. 

All the positive cultures were confirmed as *Mtb* with the MPT-64 antigen test, and could be visualized as acid-fast bacilli (AFB) following Ziehl–Neelsen staining and microscopy. We confirmed that the paired LJ-grown and lipid-grown isolates, as well as the glycerol-grown and lipid-grown isolates of samples #1 and #6, were identical and did not include mixed strains by comparing whole-genome sequences (Supplementary Table S1). 

### 2.4. Gycerol Sensitivity 

**Figure 2:**
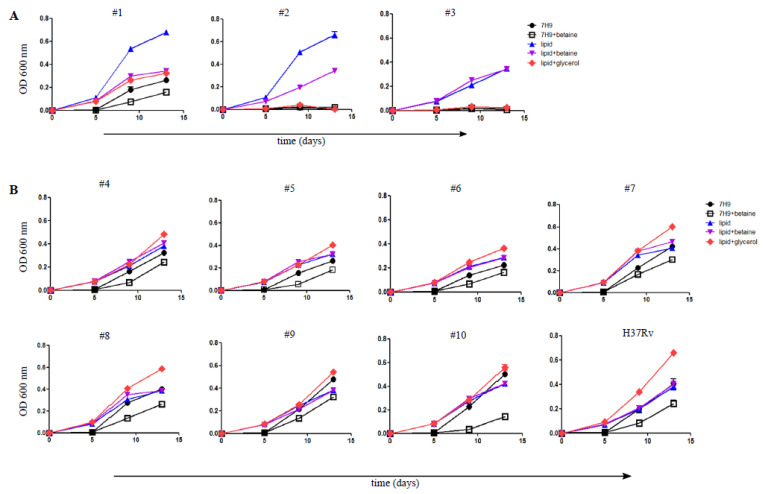
Carbon source culture assay. Growth kinetics of ten lipid-grown strains after transfer to lipid medium in the absence (blue line) or presence of betaine (purple line); glycerol-containing 7H9 in the absence (black line with closed circles) or presence of betaine (black line with open squares); lipid medium supplemented with glycerol (red lines). (**A**) Top panel shows glycerol-sensitive strains. (**B**) Bottom panel shows lipid-resuscitated strains and control H37Rv. Panels (**A**,**B**) were performed in parallel, so the H37Rv control can be compared in both panels. Points indicate the mean of triplicate cultures from a single experiment. Error bars representing standard deviation are plotted for each measurement; when not visible the error bars are smaller than the data point.

To further examine the growth phenotype and determine if failure to grow in glycerol was a heritable trait, we transferred the isolates that grew in the lipid broth into media of various compositions; glycerol-rich 7H9, lipid media, or lipid media supplemented with glycerol. To ensure that the carbon sources that were available to the bacteria were defined, Tween80 was replaced with the non-metabolizable detergent tyloxapol in these studies. We compared growth kinetics in these media and found two categories of strain. The first category consisted of glycerol-sensitive strains that grew well in lipid media, but grew relatively poorly in glycerol-containing media, despite the presence of other carbon sources in either standard 7H9 or lipid media supplemented with glycerol (Figure 2A, strains #1–#3). These strains displayed somewhat different behaviors, as glycerol slowed the growth of strain #1, but completely inhibited the growth of strains #2 and #3. This difference suggests that multiple types of mutation can produce the glycerol-sensitive phenotype. The second category consisted of seven strains that, following ‘lipid-dependent resuscitation’, grew in all the tested media. Furthermore, the addition of glycerol enhanced the growth of these strains in lipid medium (Figure 2B, strains #4–#10), as it did to the control strain, H37Rv. Additionally, supplementation of glycine betaine was no longer required to promote the culture of strains in any type of medium. Thus, among eight Sm+/Cx− sputum samples that could only be recovered in lipid-based media, we observed the following two distinct phenotypes: glycerol-sensitive strains, in which glycerol sensitivity was a hereditary trait and strains that, upon ‘lipid-dependent resuscitation’, reverted to grow in glycerol-rich media.

### 2.5. Phenotype-Associated Mutations

To investigate whether specific acquired mutations contributed to the lipid-dependent growth phenotype, we first evaluated the glycerol metabolism gene *glpK* (Rv3696c) in the ten lipid-grown strains. *GlpK* encodes glycerol-3-kinase and is essential for the utilization of glycerol. Previous studies have suggested that mutations in *glpK* may be associated with altered *Mtb* growth in glycerol [22,23]. We found a frameshift mutation caused by the insertion of +1 t in only one of the glycerol-sensitive samples (#1). This sample was one of the two samples that grew in lipid medium as well as glycerol-rich medium (Table 2), and the frameshift mutation was present in both the lipid-grown and glycerol-grown isolates.

Next, to identify other mutations, we compared the whole-genome sequences (WGS) of the ten lipid-grown strains to either (a) the WGS of the patient’s baseline sample grown on LJ, or (b) its closest related genetic match from our database of 2879 sequences from Lima, Peru that were grown in routine culture media (Supplementary Table S1). Among the three pairs of strains, which included both a baseline and a later sample, we identified 20 unique mutations, of which 16 were non-synonymous single-nucleotide polymorphisms (SNPs) (Table 3). No single mutation occurred in more than one sample. In one sample of the strains that grew in lipid and glycerol media, three mutations (*echA20*, Rv1410c, Rv1287) were detected in the lipid-grown isolate, but not in the glycerol-grown isolate. Comparing each strain to its closest genetic relative yielded a larger number of potential genetic variants (Table 4), but also failed to identify a clear association with culture phenotype.

We compared the WGS of the seven isolates for which we did not have a clinical paired strain to the closest matching strain identified in our database (to rule out lineage-associated polymorphisms). Three of the samples differed by fewer than three SNPs (Table 4, Supplementary Table S2)—notably, these had been obtained from patients who did not come from the same household, family or district of Lima, and had been collected over a period of four months. These three differed from the “closest matching isolate” in the database by >100 SNPs, of which 41 were non-synonymous (Table 4; other SNP differences and synonymous SNPs in Supplementary Table S2). None of the six non-synonymous SNPs that were identified between the four remaining pairs were present in more than one sample (Table 4).

We used STRING (version 11.0, https://string-db.org (accessed on 12 September 2019). [24]) to assess protein–protein interactions and functional enrichment among the genes in which we identified SNPs, but found no functional enrichment or protein–protein interactions (PPI enrichment *p*-values of 0.88 and 0.69, respectively).

We next determined the frequency of these SNPs in our database of WGS from Lima, Peru (*n* = 2879; Table 4, Supplementary Table S3). None occurred in >1% of the samples; *phoR* A279V/T was found in 17 samples, *fadD18* C17R in 10, Rv0846 F446V in four, *truB* A105T in two, and *PyrG* H264Y in one (Table 4, Supplementary Table S3). We noted that all patients with this *phoR* mutation were diagnosed with MDR. Thus, these SNPs are very rare in samples grown in conventional media. 

### 2.6. Drug Resistance 

**Figure 3:**
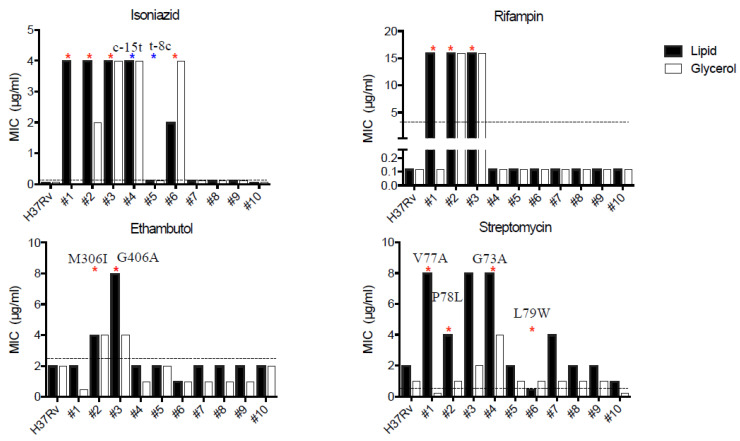
MIC results of lipid-grown strains. MIC assay performed in lipid medium (black bars) and glycerol-rich medium (white bars). * indicates presence of drug-associated mutations, as follows: INH katG[S315T] (red), fabG1 (blue; details depicted in figure); RIF rpoB[S450L]; EMB embB; SM gidB (amino acid substitutions depicted in figure). Dashed line shows WHO critical concentration in 7H9. EMB: ethambutol; INH: isoniazid; MIC: minimal inhibitory concentration; RIF: rifampin; SM: streptomycin.

We next measured the minimal inhibitory concentrations (MICs) for selected drugs in strains grown on both lipid and glycerol media. The three glycerol-sensitive isolates were resistant to INH, rifampin (RIF), and streptomycin (SM) (Figure 3). This phenotype is consistent with the clinical course of patients corresponding to isolates #1 and #2 (Figure 1). We did not observe noticeable differences between the MIC in the lipid compared with the glycerol media. WGS confirmed the presence of DR-associated mutations in samples from the three patients in whom phenotypic drug resistance had been detected via pDST at baseline (Figure 1 and Figure 3, Table 2 and Table 3). We also detected DR-associated mutations in the lipid-grown samples from three of seven isolates that initially were Cx− in LJ. 

## 3. Discussion

LJ media was developed to selectively culture *Mtb* in 1931–1932 [25,26], and this glycerol-based medium remains a primary method for recovering *M. tuberculosis* from clinical specimens almost a century later. Despite this remarkable history, LJ culture fails to detect viable *Mtb* in a significant fraction of specimens from TB patients, limiting prompt diagnosis and drug susceptibility testing. Consistent with *Mtb*’s known preference for non-glycolytic sources during infection, we found that lipid-based media effectively fostered the growth of *Mtb* from a series of AFB+ sputum samples that were not culturable in glycerol-based LJ or 7H9 media [13,14,15]. Analysis of the recovered strains demonstrated that both genetic and non-genetic mechanisms contribute to the observed differential capturability, and suggested that this phenotype may be associated with drug resistance. 

After recovery in lipid media, a number of our initially Cx− strains recovered the ability to grow in glycerol-based 7H9. Thus, for these strains, the inability to initially grow in LJ or 7H9 represented a non-heritable adaptation to the pulmonary environment. This observation is generally consistent with previous studies describing the differential culturability of *Mtb* based on the growth condition. Several groups have shown that subpopulations of *Mtb* that do not grow in routine culture can be resuscitated in the presence of culture filtrate that contains *Mtb*-secreted factors [7,8]. In these studies, the addition of culture filtrate to routine media increased the numbers of *Mtb* bacilli that were detected in the Cx+ sputum samples. Although the mechanism by which culture filtrate stimulates the growth of otherwise un-culturable bacilli is unknown, some investigators have proposed that fatty acids may be a resuscitating factor [27]. Notably, fatty acids were the main component of our lipid-based medium. While our study focused only on Cx− samples, it is possible that the bacilli we were able to culture have a similar phenotype to the differentially detectable *Mtb* described previously.

In contrast to these reversable adaptations, a subset of Cx− strains, recovered in lipid media, demonstrated a heritable glycerol-sensitive phenotype. These strains were unable to grow in the glycerol-containing 7H9, despite the presence of other carbon sources in the media. This phenotype is unrelated to the common drug-resistance-associated frameshift mutations in the *glpK* gene. Not only were *glpK* mutations absent from two of the three glycerol-sensitive strains, but the loss of GlpK only prevents glycerol catabolism and does not eliminate growth on the other carbon sources that are present in 7H9 [22]. While the heritable glycerol-sensitive phenotype was differentiable from *glpK* mutants, it is notable that both this phenotype and *glpK* frameshift mutations are associated with MDR strains. This association suggests that drug exposure may select for a variety of glycerol-related mutations, perhaps because they reduce drug efficacy in a similar manner as *glpK* frameshifts.

Our study has a number of limitations. These include the relatively small sample size and the lack of matched strains for genetic analysis. In addition, although some studies have shown that the storage time does not affect the recovery rate of Mtb from sputum after freezing [28], others suggest a possible loss of bacteria after cryopreservation of sputum, and thus, we cannot rule out the possibility that long-term storage and freezing altered the metabolic function of the organisms [29]. Nonetheless, the ability to recover *Mtb* from persistently Cx− samples indicates that, despite the long history and utility of standard glycerol-based media, new formulations could improve diagnosis. Perhaps more concerning is the novel glycerol-sensitive phenotype described in this study, as this trait would be expected to specifically impede the culture-based diagnosis and subsequent DST of drug-resistant *Mtb* strains. Future studies, with larger and more diverse sample sets, will be necessary to address these possibilities. 

## 4. Methods

### 4.1. Ethics

The study protocol was approved by the Harvard University Institutional Review Board (Ref. No. 19331) and by the research ethics committee of the National Institute of Health of Peru.

### 4.2. Carbon-Source Growth Experiments

Frozen, NALC-OH-decontaminated sputum samples were thawed and 30–60 μL of sample was cultivated in (1) glycerol-rich medium: Middlebrook 7H9 supplemented with 0.2% glycerol, 0.05% Tween80 and albumin–dextrose–catalase solution; (2) lipid-rich/glycerol-poor medium. Because commercial media using lipids as the sole carbon source are not available, and because commercial lipid-supplemented media may include free fatty acids and cholesterol-metabolized C3 intermediates, which harbor some toxicity to bacterial growth [30], we designed a neutral lipid media with variable carbon chain numbers of fatty acids, from medium to long chains, some of which can be directly utilized for lipid synthesis in the cells, and others for beta-oxidation. A stable lipid emulsion was prepared from 5 g coconut oil (Sigma-Aldrich cat# C175) mixed in 25 mL 20% Tween80 and added to 10X AC (bovine serum albumin and catalase) solution at 1:100 (vol/vol). The medium was finalized by addition of sodium phosphate buffer solution (pH 6.5) at 50 mM and enriched with 0.05% Tween80, and 1x albumin/catalase solution. Thin-layer chromatography revealed the composition of the lipid media to be mainly free fatty acids, mono-, di- and triacylglyceride.

Both the media were supplemented with 2.5 μg/mL amphotericin B and 0.1 mg/mL trimethoprim to prevent contamination from other microorganisms. Liquid cultures were incubated under gentle shaking at 37 °C. In case of delayed growth, 1 mM glycine betaine was added into the cultures after four weeks. Optic density (OD_600_) was measured over time and presence of *Mtb* was confirmed by MPT-64 antigen detection (Standard Diagnostics Inc.) and Ziehl–Neelsen staining.

For the glycerol-toxicity culture assay, *Mtb* was grown in 7H9 medium containing 0.5% glycerol, fatty acid-free albumin/catalase and 0.05% tyloxapol. OD_600_ was measured over time.

To prepare stocks, isolates from lipid-grown strains were cultured on lipid agar and stored in lipid medium supplemented with 1% DMSO. 7H9–glycerol-grown strains were cultivated on 7H10 agar and preserved in 10% glycerol-containing 7H9 medium. All stocks were stored at −80 °C.

### 4.3. Phenotypic Drug Susceptibility Tests 

MICs of the isolates were determined in lipid-rich/glycerol-poor media or glycerol-rich media described above. Strains were inoculated into both media at OD 0.0008 in a Sensititre MYCOTB MIC DST plate. Drug dilutions were prepared in DMSO and added to the microplate culture with a final concentration of 2% DMSO. Two-fold serial dilutions of drugs were prepared with the following maximal concentrations (mg/L): INH (64), RIF (4), SM (32), EMB (16). Thereafter, the cultures were incubated for 15 days at 37 °C. Viability was determined using a fluorimeter (BMG Labtech). We measured MIC_90_ for all other drugs. The reference H37Rv (ATCC 27294) MTB strain was included as a control in the experiments.

### 4.4. DNA Extraction 

Genomic DNA was extracted from individual colonies for each isolate grown on 7H10 or lipid agar plates using a standard CTAB method [31]. In brief, harvested cultures were suspended in trypsin-EDTA buffer (pH 8) and denatured by heating at 80 °C. Then cells were lysed with lysozyme (10 mg/mL), proteinase K (10 mg/mL), and sodium dodecyl sulfate (10%). Next, NaCl and *N*-acetyl, *N*,*N*,*N*-cetyltrimethyl ammonium bromide (CTAB) were added. DNA was extracted and purified with chloroform/isoamyl alcohol (24:1), isopropanol, and 70% ethanol. 

### 4.5. Sequencing

Samples were prepared for sequencing using the standard Illumina whole-genome sample preparation kit (Illumina, Inc.; San Diego, CA, USA) and sequenced on an Illumina 2500 or MiSeq instrument. Paired-end reads with a read length of 125–150 bp were collected. The mean depth of coverage ranged from 52 to 94×. Genome sequences for the isolates were assembled using a comparative assembly approach [32]. Reads were mapped to the *Mtb* H37Rv (NC_000962.2) reference genome using BWA v0.7.12 [33], and insertions and deletions (indels) of size 1 bp–20 kb were identified using local contig building. Polymorphisms were identified by aligning each genome to the sequence of H37Rv (using MUMmer v3.20 [34]) and tabulating single-nucleotide polymorphisms (SNPs) and indels according to the following criteria: coverage by at least 10 reads, and not heterogeneous (≥70% conversion to the nonreference nucleotide). SNPs in repetitive regions were also filtered out (defined as sites for which an overlapping 35 bp window matched a sequence elsewhere in the genome with at most 2 mismatches). GlpK mutations were identified as reported previously [22].

## Figures and Tables

**Table 1:** Numbers of patients in our cohort (*n* = 5192) with a culture-negative sputum sample at baseline and/or smear-positive/culture-negative sample post treatment, stratified by AFB smear results. AFB: acid-fast bacilli; tx: treatment.

AFB	Baseline (*n*)	Post Tx Initiation (*n*)
−	787	*Not relevant*
1–9 (scanty)	223	564
+	50	143
++	16	32
+++	15	22
Total	1091	760

**Table 2:** Phenotypic DST (pDST)) results and treatment regimens for the 15 patients selected for the study.

Patient #	Resistance Profile Measured with pDST	Time pDST Sample Collection	Treatment Regimen
1 ^a^	INH, RIF, EMB, SM, CM, KM	Study enrollment ^b^	CPX, EMB, ETH, KM, PZA (months 0–4)
			AMK, EMB, LEVO (months 9–18)
2 ^a^	INH, RIF	Study enrollment	AMK, CS, EMB, ETH, LFX, PZA
	INH, RIF, EMB, PZA, SM	2 months of treatment	
3	-	-	unknown
4	-	-	INH, RIF, PZA, EMB
5	-	-	unknown
6	-	-	INH, RIF, PZA, EMB
7	-	-	INH, RIF, PZA, EMB
8	-	-	INH, RIF, PZA, EMB
9	-	-	INH, RIF, PZA, EMB
10	-	-	INH, RIF, PZA, EMB
11	-	-	INH, RIF, PZA, EMB
12			INH, RIF, PZA, EMB
13	Drug susceptible	Study enrollment	CPX, CS, EMB, ETH
14	-	-	INH, RIF, PZA, EMB
15 ^a^	INH, RIF, EMB, SM	Study enrollment	CS, EMB, ETH, LFX, PZA

^a^ Patient received MDR-TB diagnosis at time of study enrollment. ^b^ Patient 1 was on treatment for 19 months prior to enrollment. AMK: amikacin; CM: capreomycin; CPX: ciprofloxacin; CS: cycloserine; pDST: phenotypic drug-sensitivity testing; EMB: ethambutol; ETH: ethionamide; KM: kanamycin; LFX: levofloxacin; PZA: pyrazinamide; INH: isoniazid; RIF: rifampin; SM: streptomycin.

**Table 3:** Carbon source culture results and analysis of drug-resistance (DR)-associated SNPs for 15 Sm+/Cx− samples. MDR-TB: multidrug-resistant tuberculosis; OD: optic density; SNP: single-nucleotide polymorphism.

Patient #	Carbon Source Culture Data (OD (Days))		DR-Associated SNPs ^a^	Growth Phenotype
	Glycerol medium	Lipid medium		
1 ^a^	0.22 (28)	0.31 (28)	*rpoB*[S450L]; *katG*[S315T]; *gidB*[V77A]	Glycerol-sensitive
2 ^a^	-	0.14 (35)	*rpoB*[S450L]; *katG*[S315T]; *embB*[M306I]; *gidB*[P78L]	Glycerol-sensitive
3	-	0.16 (49)	*rpoB*[S450L]; *katG*[S315T]; *embB* [G406A]; *pncA* [A172T]	Glycerol-sensitive
4 ^b^	-	0.24 (28)	*katG*[S315T]; *fabG1* * c-15t; *ethA*[S399 *]; *gidB[*G73A]	Lipid-dependent resuscitation
5	-	0.12 (28)	*fabG1* * t-8c	Lipid-dependent resuscitation
6	0.17 (35)	0.28 (28)	*katG*[S315T]; *gidB*[L79W]	Lipid-dependent resuscitation
7	-	0.15 (50) ^c^	no DR-SNPs	Lipid-dependent resuscitation
8 ^b^	-	0.23 (50) ^c^	no DR-SNPs	Lipid-dependent resuscitation
9	-	0.25 (62) ^c^	no DR-SNPs	Lipid-dependent resuscitation
10	-	0.11 (33)	no DR-SNPs	Lipid-dependent resuscitation
11		-		No lipid-dependent growth
12	-	-		No lipid-dependent growth
13	-	-		No lipid-dependent growth
14	-	-		No lipid-dependent growth
15 ^a^	-	-		No lipid-dependent growth

^a^ Patient received MDR-TB diagnosis at moment of study enrollment. ^b^ Analysis of well-known DR-associated SNPs in *embB*, *ethA*, *gid*, *gyrA*, *inhA*, inter-*embC*-*embA*, *KatG*, *pncA*, *rpoB*, *rpsL*, *rrs*, up-eis, upstream *fabG1*. ^c^ growth in lipid medium initiated in the presence of glycine betaine.

**Table 4:** SNP differences in lipid-grown Sm+/Cx− samples, collected at least a year later following TB treatment, compared to paired patient baseline samples, collected at study enrollment.

#	Annotation	Gene Name	Mutation	Mutation Type	Product	Classification	Samples in Database with a.a. Variant (N)
1	Rv1599*	*hisD*	A259V	non-synonymous	Probable histidinol dehydrogenase HisD (HDH)	intermediary metabolism and respiration	0
1	Rv3854c ^a^	*ethA*	L48F	non-synonymous	Monooxygenase EthA	intermediary metabolism and respiration	0
1	Rv0092 ^a^	*ctpA*	S678P	non-synonymous	Cation transporter P-type ATPase a CtpA	cell wall and cell processes	0
1	Rv2576c	*-*	T103I	non-synonymous	Possible conserved membrane protein	cell wall and cell processes	0
2	Rv3151	*nuoG*	L40P	non-synonymous	Probable NADH dehydrogenase I (chain G) NuoG (NADH-ubiquinone oxidoreductase chain G)	intermediary metabolism and respiration	0
2	Rv2931 ^a^	*ppsA*	P955H	non-synonymous	Phenolpthiocerol synthesis type-I polyketide synthase	lipid metabolism	0
2		g > a Rv2304c-Rv2305		intergenic			-
6	Rv3513c	*fadD18*	C17R	non-synonymous	Probable fatty-acid-CoA ligase FadD18 (fragment) (fatty-acid-CoA synthetase) (fatty-acid-CoA synthase)	lipid metabolism	10
6	Rv3550 ^a^	*echA20*	G109A	non-synonymous	Probable enoyl-CoA hydratase EchA20	lipid metabolism	0
6	Rv0758	*phoR*	A279T	non-synonymous	Possible two component system response sensor kinase membrane associated PhoR	regulatory proteins	17 (15 patients)
6	Rv2793c	*truB*	A105T	non-synonymous	Probable tRNA pseudouridine synthase B TruB	information pathways	2
6	Rv1410c ^b^	-	Q301H	non-synonymous	Aminoglycosides/tetracycline-transport integral membrane protein	cell wall and cell processes	0
6	Rv1287 ^b^	-	V43I	non-synonymous	Conserved hypothetical protein	conserved hypotheticals	0
6	Rv2402	-	G600G	synonymous	Conserved protein	conserved hypotheticals	-
6	Rv3087	-	23bp del aa 403	Indel (frameshift)	Possible triacylglycerol synthase (diacylglycerol acyltransferase)	lipid metabolism	-
6	Rv1959c	*parE1*	Q12H	non-synonymous	Possible toxin ParE1	virulence, detoxification, adaptation	0
6	Rv3861	-	R63H	non-synonymous	Hypothetical protein	conserved hypotheticals	0
6	Rv0326		D101D	synonymous	Hypothetical protein	Unknown	-
6	Rv1721c	*VapB12*	D48E	non-synonymous	Possible antitoxin VapB12	virulence, detoxification, adaptation	0
6	Rv0236c ^c^	*aftD*	F21L	non-synonymous	Possible arabinofuranosyltransferase AftD	cell wall and cell processes	0

^a^ Amino acid substitutions are identified in reference to the consensus sequence of the H37RV strain. ^b^ Mutation was detected in lipid-grown strain, not in the glycerol-grown strain. ^c^ Mutation was detected in glycerol-grown strain, not in the lipid-grown strain.

## Data Availability

The data reported in this study are available in the text of this article and in Supplementary Tables S1–S3.

## Supplementary Materials

The following are available online at https://www.mdpi.com/article/10.3390/jcm10153249/s1, Table S1: Lineage of lipid-grown and glycerol-grown strains and the reference strains they were compared to in whole genome sequencing SNP analysis **Table S2**: All SNP differences between lipid-grown Sm+/Cx− samples and closest genetic match. **Table S3**: SNP differences between lipid-grown Sm+/Cx− samples and closest genetic match within Peru cohort that could be grown on glycerol.

## Abbreviations

AFB	acid-fast bacilli
AMK	amikacin
CM	capreomycin
CPX	ciprofloxacin
CS	cycloserine
Cx	culture
DR	drug resistant
DS	drug susceptible
EMB	ethambutol
ETH	ethionamide
HHC	household contacts
INH	Isoniazid
KM	kanamycin
LFX	levofloxacin
LJ	Lowenstein–Jensen medium
LTFU	lost to follow-up
MDR	multidrug-resistant TB
MGIT	mycobacterial growth incubator tube
MIC	minimal inhibitory concentrations
monoR	mono drug resistant
*Mtb*	*Mycobacterium tuberculosis*
OD	optic density
(p)DST	(phenotypic) drug-sensitivity testing
PZA	pyrazinamide
RIF	rifampicin
Sm	smear
SM	streptomycin
SNP	single-nucleotide polymorphisms
TB	tuberculosis
Tx	treatment
WGS	whole-genome sequencing
