# Supplementary material for: Characterization of Drug-Resistant Lipid-Dependent Differentially Detectable Mycobacterium tuberculosis
Source: J Clin Med. 2021 Jul 23;10(15):3249. doi: 10.3390/jcm10153249 (PMC8348819; doi:10.3390/jcm10153249)
Supplement: Supplementary file 1 [file jcm-10-03249-s001.zip › suppl table 1_July6.pdf]

Supplementary table 1. lineage of lipid-grown and glycerol-grown strains and the reference strains they were compared to in whole genome sequencing SNP analysis

	Patient #	Lipid-grown	Glycerol-grown	Reference
Paired patient samples	1	L4.1	L4.1.2	L4.1.2
	2	L4.3		L4.3
	6	L4.x	L4.x	L4.x
Closest genetic match	3	L4.8		L4.8
	4	L4.1		L4.1.2
	5	L4.1		L4.1.2
	7	L4.1		L4.1.2
	8	L4.1		L4.1.2
	9	L4.1		L4.1.2
	10	L2		L2
