# Supplementary material for: Characterization of Drug-Resistant Lipid-Dependent Differentially Detectable Mycobacterium tuberculosis
Source: J Clin Med. 2021 Jul 23;10(15):3249. doi: 10.3390/jcm10153249 (PMC8348819; doi:10.3390/jcm10153249)
Supplement: Supplementary file 1 [file jcm-10-03249-s001.zip › suppl table 2_July6.pdf]

**Supplementary table 2**. All SNP differences between lipid-grown Sm+/Cx- samples and closest genetic match.

| Sample | Annotation | Gene Name | Mutation | Mutation type | Product | Mycobrowser classification |
|---|---|---|---|---|---|---|
| 7 | Rv0145 | - | E147E | synonymous | Possible S-adenosylmethionine-dependent methyltransferase | lipid metabolism |
| 10 | g>a between Rv3312c and Rv3312A | | | intergenic | | |
| 10 | ~163kb dup Rv3180c-Rv3324c | | | duplication | | |
| 10 | Rv0998 | pat | D276D | synonymous | Conserved hypothetical protein | conserved hypotheticals |
| 10 | Rv1922 | | G228A | non-synonymous | Probable conserved lipoprotein | cell wall and cell processes |
| 7,8,9 | Rv0974c | accD2 | +1 bp | indel | Probable acetyl-/propionyl-CoA carboxylase (beta subunit) AccD2 | lipid metabolism |
| 7,8,9 | Rv3919c | gid | 4402868 +G | indel | Probable glucose-inhibited division protein B Gid | cell wall and cell processes |
| 7,8,9 | Rv0111 | - | I628I | synonymous | Possible transmembrane acyltransferase | intermediary metabolism and respiration |
| 7,8,9 | Rv0224c | - | L50L | synonymous | Possible methyltransferase (methylase) | intermediary metabolism and respiration |
| 7,8,9 | Rv0286 | PPE4 | A166A | synonymous | PPE family protein PPE4 | PE/PPE |
| 7,8,9 | Rv0338c | - | K664K | synonymous | Probable iron-sulfur-binding reductase | intermediary metabolism and respiration |
| 7,8,9 | Rv0878c | PPE13 | 981911 -2bp | indel | PPE family protein PPE13 | PE/PPE |
| 7,8,9 | Rv0928 | pstS3 | G212G | synonymous | Periplasmic phosphate-binding lipoprotein PstS3 (PBP-3) (PstS3) (PHOS1) | cell wall and cell processes |
| 7,8,9 | Rv1190 | - | 1339588 -2bp | indel | Conserved hypothetical protein | conserved hypotheticals |
| 7,8,9 | Rv1226c | - | L414L | synonymous | Probable transmembrane protein | cell wall and cell processes |
| 7,8,9 | Rv1257c | - | V404V | synonymous | Probable oxidoreductase | intermediary metabolism and respiration |
| 7,8,9 | Rv1421 | - | A5A | synonymous | Conserved protein | conserved hypotheticals |
| 7,8,9 | Rv1488 | | +2bp | indel | Possible exported conserved protein | cell wall and cell processes |
| 7,8,9 | Rv1562c | treZ | Y195Y | synonymous | Maltooligosyltrehalose trehalohydrolase TreZ | virulence, detoxification, adaptation |
| 7,8,9 | Rv1941 | - | A226A | synonymous | Probable short-chain type dehydrogenase/reductase | intermediary metabolism and respiration |
| 7,8,9 | Rv1979c | - | A298A | synonymous | Possible conserved permease | cell wall and cell processes |
| 7,8,9 | Rv2011c | - | A123A | synonymous | Conserved hypothetical protein, probable transcription repressor. | regulatory proteins |
| 7,8,9 | Rv2221c | glnE | D865D | synonymous | Glutamate-ammonia-ligase adenylyltransferase GlnE (glutamine-synthetase adenylyltransferase) | intermediary metabolism and respiration |

| | | | | | | |
|---|---|---|---|---|---|---|
| 7,8,9 | Rv2241 | aceE | T318T | synonymous | Pyruvate dehydrogenase E1 component AceE (pyruvate decarboxylase) (pyruvate dehydrogenase) (pyruvic dehydrogenase) | intermediary metabolism and respiration |
| 7,8,9 | Rv2248 | - | 2527359 -1bp | indel | Conserved hypothetical protein | conserved hypotheticals |
| 7,8,9 | Rv2426c | - | I262I | synonymous | Conserved hypothetical protein | conserved hypotheticals |
| 7,8,9 | Rv2794c | pptT | L74L | synonymous | Phosphopantetheinyl transferase PptT (CoA:APO-[ACP]pantetheinephosphotransferase) (CoA:APO-[acyl-carrier protein]pantetheinephosphotransferase) | lipid metabolism |
| 7,8,9 | Rv2941 | fadD28 | A52A | synonymous | Fatty-acid-AMP ligase FadD28 (fatty-acid-AMP synthetase) (fatty-acid-AMP synthase) | lipid metabolism |
| 7,8,9 | Rv3030 | - | L135L | synonymous | Conserved protein | conserved hypotheticals |
| 7,8,9 | Rv3234c | tgs3 | 3601577 +G | indel | Putative triacylglycerol synthase (diacylglycerol acyltransferase) Tgs3 | lipid metabolism |
| 7,8,9 | Rv3296 | lhr | S683S | synonymous | Probable ATP-dependent helicase Lhr (large helicase-related protein) | information pathways |
| 7,8,9 | Rv3303c | lpdA | 3680983 +ATCG | indel | NAD(P)H quinone reductase LpdA | intermediary metabolism and respiration |
| 7,8,9 | Rv3578 | arsB2 | S235S | synonymous | Possible arsenical pump integral membrane protein ArsB2 | cell wall and cell processes |
| 7,8,9 | Rv3663c | dppD | L414L | synonymous | Probable dipeptide-transport ATP-binding protein ABC transporter DppD | cell wall and cell processes |
| 7,8,9 | Rv3688c | - | A66A | synonymous | Conserved protein | conserved hypotheticals |
| 7,8,9 | Rv3785 | - | T139T | synonymous | Hypothetical protein | conserved hypotheticals |
| 7,8,9 | Rv3792 | aftA | T478T | synonymous | Arabinofuranosyltransferase AftA | cell wall and cell processes |
| 7,8,9 | Rv3809c | glf | G235G | synonymous | UDP-galactopyranose mutase Glf (UDP-GALP mutase) (NAD+-flavin adenine dinucleotide-requiring enzyme) | cell wall and cell processes |
| 7,8,9 | Rv3862c | whiB6 | R17R | synonymous | Possible transcriptional regulatory protein WhiB-like WhiB6 | conserved hypotheticals |
| 7,8,9 | Rv3899c | - | -GGAG downstream of Rv3899c | intergenic | Conserved hypothetical protein | conserved hypotheticals |
| 7,8,9 | | | | intergenic | | |

| | | | | | | |
|---|---|---|---|---|---|---|
| | 88297 T>C (7 bp upstream of Rv0078A/-) | | | | | |
| 7,8,9 | 297284 T>G (19 bp upstream of Rv0246/-) | | | intergenic | | |
| 7,8,9 | 998804 A>G (64 bp upstream of Rv0892/-) | | | intergenic | | |
| 7,8,9 | 1072491 G>A (30 bp downstream of Rv0955/-) | | | intergenic | | |
| 7,8,9 | 1475872 T>G (4 bp downstream of Rv1314c/-) | | | intergenic | | |
| 7,8,9 | 2003092 T>G (277 bp upstream of Rv1762c/-) | | | intergenic | | |
| 7,8,9 | 2062392 T>C (542 bp downstream of Rv1811/mgtC) | | | intergenic | | |
| 7,8,9 | 2581334 G>T (46 bp upstream of Rv2311/-) | | | intergenic | | |
| 7,8,9 | Rv0069c | sdaA | G417D | non-synonymous | Probable L-serine dehydratase SdaA (L-serine deaminase) (SDH) (L-SD) | intermediary metabolism and respiration |
| 7,8,9 | Rv0411c | glnH | P39T | non-synonymous | Probable glutamine-binding lipoprotein GlnH (GLNBP) | cell wall and cell processes |
| 7,8,9 | Rv0479c | | M318V | non-synonymous | Probable conserved membrane protein | cell wall and cell processes |
| 7,8,9 | Rv0510 | hemC | V194A | non-synonymous | Probable porphobilinogen deaminase HemC (PBG) (hydroxymethylbilane synthase) (HMBS) (pre-uroporphyrinogen synthase) | intermediary metabolism and respiration |
| 7,8,9 | Rv0512 | hemB | F52S | non-synonymous | Probable delta-aminolevulinic acid dehydratase HemB (porphobilinogen synthase) (ALAD) (ALADH) | intermediary metabolism and respiration |
| 7,8,9 | Rv0701 | rplC | V137A | non-synonymous | 50S ribosomal protein L3 RplC | information pathways |
| 7,8,9 | Rv0726c | | V118L | non-synonymous | Possible S-adenosylmethionine-dependent methyltransferase | lipid metabolism |
| 7,8,9 | Rv0727c | fucA | M101V | non-synonymous | Possible L-fuculose phosphate aldolase FucA (L-fuculose-1-phosphate aldolase) | intermediary metabolism and respiration |
| 7,8,9 | Rv1018c | glmU | K363N | non-synonymous | Probable UDP-N-acetylglucosamine pyrophosphorylase GlmU | cell wall and cell processes |

| | | | | | | |
|---|---|---|---|---|---|---|
| 7,8,9 | Rv1061 | | V252A | non-synonymous | Conserved protein | conserved hypotheticals |
| 7,8,9 | Rv1187 | rocA | R217Q | non-synonymous | Probable pyrroline-5-carboxylate dehydrogenase RocA | intermediary metabolism and respiration |
| 7,8,9 | Rv1217c | | S327G | non-synonymous | Probable tetronasin-transport integral membrane protein ABC transporter | cell wall and cell processes |
| 7,8,9 | Rv1253 | deaD | G326D | non-synonymous | Probable cold-shock DeaD-box protein A homolog DeaD (ATP-dependent RNA helicase dead homolog) | information pathways |
| 7,8,9 | Rv1256c | cyp130 | P171Q | non-synonymous | Probable cytochrome P450 130 Cyp130 | intermediary metabolism and respiration |
| 7,8,9 | Rv1280c | oppA | S65A | non-synonymous | Probable periplasmic oligopeptide-binding lipoprotein OppA | cell wall and cell processes |
| 7,8,9 | Rv1358 | | L423F | non-synonymous | Probable transcriptional regulatory protein | regulatory proteins |
| 7,8,9 | Rv1550 | fadD11 | R501G | non-synonymous | Probable fatty-acid-CoA ligase FadD11 (fatty-acid-CoA synthetase) (fatty-acid-CoA synthase) | lipid metabolism |
| 7,8,9 | Rv1630 | rpsA | E45K | non-synonymous | 30S ribosomal protein S1 RpsA | information pathways |
| 7,8,9 | Rv1908c | katG | L351W | non-synonymous | Catalase-peroxidase-peroxynitritase T KatG | virulence, detoxification, adaptation |
| 7,8,9 | Rv2066 | cobI | A181V | non-synonymous | Probable bifunctional protein, CobI-COBJ fusion protein: S-adenosyl-L-methionine-precorrin-2 methyl transferase + precorrin-3 methylase | intermediary metabolism and respiration |
| 7,8,9 | Rv2198c | mmpS3 | D92E | non-synonymous | Probable conserved membrane protein MmpS3 | cell wall and cell processes |
| 7,8,9 | Rv2318 | uspC | A150V | non-synonymous | Probable periplasmic sugar-binding lipoprotein UspC | cell wall and cell processes |
| 7,8,9 | Rv2447c | folC | A153E | non-synonymous | Probable folylpolyglutamate synthase protein FolC (folylpoly-gamma-glutamate synthetase) (FPGS) | intermediary metabolism and respiration |
| 7,8,9 | Rv2484c | | S116P | non-synonymous | Possible triacylglycerol synthase (diacylglycerol acyltransferase) | lipid metabolism |
| 7,8,9 | Rv2595 | VapB40 | R46G | non-synonymous | Possible antitoxin VapB40 | virulence, detoxification, adaptation |
| 7,8,9 | Rv2942 | mmpL7 | F180V | non-synonymous | Conserved transmembrane transport protein MmpL7 | cell wall and cell processes |
| 7,8,9 | Rv2950c | fadD29 | G536R | non-synonymous | Fatty-acid-AMP ligase FadD29 (fatty-acid-AMP synthetase) (fatty-acid-AMP synthase) | lipid metabolism |
| 7,8,9 | Rv2953 | | R3P | non-synonymous | Enoyl reductase | lipid metabolism |

| | | | | | | |
|---|---|---|---|---|---|---|
| 7,8,9 | Rv3401 | | I83V | non-synonymous | Conserved protein | intermediary metabolism and respiration |
| 7,8,9 | Rv3499c | mce4A | R54M | non-synonymous | Mce-family protein Mce4A | virulence, detoxification, adaptation |
| 7,8,9 | Rv3685c | cyp137 | I235L | non-synonymous | Probable cytochrome P450 137 Cyp137 | intermediary metabolism and respiration |
| 7,8,9 | Rv3866 | espG1 | G112V | non-synonymous | ESX-1 secretion-associated protein EspG1 | cell wall and cell processes |
| 7,8,9 | 2605818 T>G (1 bp upstream of Rv2333c/-) | | | intergenic | | |
| 7,8,9 | 2866534 T>G (151 bp downstream of Rv2549c/-) | | | intergenic | | |
| 7,8,9 | 2931088 G>A (62 bp upstream of Rv2606c/-) | | | intergenic | | |
| 7,8,9 | 3024751 T>C (20 bp upstream of Rv2716/-) | | | intergenic | | |
| 7,8,9 | 3631459 T>C (164 bp upstream of Rv3261/fbiA) | | | intergenic | | |
| 7,8,9 | 3741534 C>T (47 bp upstream of Rv3347c/PPE55) | | | intergenic | | |
| 7,8,9 | 3927970 G>A (6 bp downstream of Rv3507/PE_PGRS53) | | | intergenic | | |
| 7,8,9 | 4185374 G>A (34 bp upstream of Rv3739c/PPE67) | | | intergenic | | |
| 7,8,9 | 4333368 T>C (24 bp upstream of Rv3862c/whiB6) | | | intergenic | | |
