# Supplementary material for: Characterization of Drug-Resistant Lipid-Dependent Differentially Detectable Mycobacterium tuberculosis
Source: J Clin Med. 2021 Jul 23;10(15):3249. doi: 10.3390/jcm10153249 (PMC8348819; doi:10.3390/jcm10153249)
Supplement: Supplementary file 1 [file jcm-10-03249-s001.zip › suppl table 3_July6.pdf]

Supplementary Table 3. SNP differences between lipid-grown Sm+/Cx- samples and closest genetic match within Peru cohort that could be grown on glycerol.

#	Annotation	Gene Name	Mutation	Product	Classification	Samples in database with a.a. variant (N)
3	Rv0656c	*vapC6*	A125V	Possible toxin VapC6	virulence, detoxification, adaptation	0
3	Rv2083		Y11C	Conserved hypothetical protein	conserved hypotheticals	0
3	Rv2281	*pitB*	A288T	Putative phosphate-transport permease PitB	cell wall and cell processes	0
4	Rv1388	*mihF*	G3S	Putative integration host factor MihF	information pathways	0
4	Rv3578	*arsB2*	G312R	Possible arsenical pump integral membrane protein ArsB2	cell wall and cell processes	0
5	Rv2066	*cobI*	Q155H	Probable bifunctional protein, CobI-COBJ fusion protein: S-adenosyl-L-methionine-precorrin-2 methyl transferase + precorrin-3 methylase	intermediary metabolism and respiration	0
7	Rv3455c	*truA*	T46K	Probable tRNA pseudouridine synthase a TruA (pseudouridylate synthase I) (pseudouridine synthase I) (uracil hydrolyase)	information pathways	0
7,8,9	Rv0070c	*glyA2*	D201N	Serine hydroxymethyltransferase GlyA2 (serine methylase 2) (SHMT 2)	intermediary metabolism and respiration	0
7,8,9	Rv0089	-	A104V	Possible methyltransferase/methylase	intermediary metabolism and respiration	0
7,8,9	Rv0338c	-	L502F	Probable iron-sulfur-binding reductase	intermediary metabolism and respiration	0
7,8,9	Rv0511	*hemD*	M475R	Probable uroporphyrin-III C-methyltransferase HemD (uroporphyrinogen III methylase) (urogen III methylase) (SUMT) (urogen III methylase) (UROM)	intermediary metabolism and respiration	0
7,8,9	Rv0675	*echA5*	A68D	Probable enoyl-CoA hydratase EchA5 (enoyl hydrase) (unsaturated acyl-CoA hydratase) (crotonase)	lipid metabolism	0
7,8,9	Rv0724	*sppA*	G55D	Possible protease IV SppA (endopeptidase IV) (signal peptide peptidase)	cell wall and cell processes	0
7,8,9	Rv0773c	*ggtA*	E467K	Probable bifunctional acylase GgtA: cephalosporin acylase (GL-7ACA acylase) + gamma-glutamyltranspeptidase (GGT)	intermediary metabolism and respiration	0
7,8,9	Rv0846c	-	F446V	Probable oxidase	intermediary metabolism and respiration	4
7,8,9	Rv0949	*uvrD1*	T600I	Probable ATP-dependent DNA helicase II UvrD1	information pathways	0
7,8,9	Rv1115	-	A128V	Possible exported protein	cell wall and cell processes	0
7,8,9	Rv1358	-	C992W	Probable transcriptional regulatory protein	regulatory proteins	0
7,8,9	Rv1392	*metK*	P106L	Probable S-adenosylmethionine synthetase MetK (mat) (AdoMet synthetase) (methionine adenosyltransferase)	intermediary metabolism and respiration	0
7,8,9	Rv1407	*fmu*	E22K	Probable Fmu protein (sun protein)	information pathways	0
7,8,9	Rv1480	-	A8T	Conserved protein	conserved hypotheticals	0
7,8,9	Rv1527c	*pks5*	Q281R	Probable polyketide synthase Pks5	lipid metabolism	0
7,8,9	Rv1639c	-	P470Q	Conserved hypothetical membrane protein	cell wall and cell processes	0
7,8,9	Rv1657	*argR*	D147N	Probable arginine repressor ArgR (AHRC)	regulatory proteins	0
7,8,9	Rv1699	*pyrG*	H264Y	Probable CTP synthase PyrG	intermediary metabolism and respiration	1
7,8,9	Rv1900c	*lipJ*	F300S	Probable lignin peroxidase LipJ	intermediary metabolism and respiration	0
7,8,9	Rv1972	-	A112V	Probable conserved Mce associated membrane protein	cell wall and cell processes	0
7,8,9	Rv2054	-	E223Q	Conserved protein	conserved hypotheticals	0

7,8,9	Rv2163c	*pbpB*	Q240R	Probable penicillin-binding membrane protein PbpB	cell wall and cell processes	0
7,8,9	Rv2189c	-	R2S	Conserved hypothetical protein	conserved hypotheticals	0
7,8,9	Rv2230c	-	S106A	Conserved protein	conserved hypotheticals	0
7,8,9	Rv2248	-	G271V	Conserved hypothetical protein	conserved hypotheticals	0
7,8,9	Rv2249c	*glpD1*	T326A	Probable glycerol-3-phosphate dehydrogenase GlpD1	intermediary metabolism and respiration	0
7,8,9	Rv2307D	-	T49K	Hypothetical protein	conserved hypotheticals	0
7,8,9	Rv2397c	*cysA1*	A167V	Sulfate-transport ATP-binding protein ABC transporter CysA1	cell wall and cell processes	0
7,8,9	Rv2510c	-	L77P	Conserved protein	conserved hypotheticals	0
7,8,9	Rv2528c	*mrr*	Q52*	Probable restriction system protein Mrr	information pathways	0
7,8,9	Rv2594c	*ruvC*	R168W	Probable crossover junction endodeoxyribonuclease RuvC (holliday junction nuclease) (holliday junction resolvase)	information pathways	0
7,8,9	Rv2640c	-	S54L	Possible transcriptional regulatory protein (probably ArsR-family)	regulatory proteins	0
7,8,9	Rv2661c	-	I107V	Hypothetical protein	conserved hypotheticals	0
7,8,9	Rv2752c	-	R481C	Conserved hypothetical protein	conserved hypotheticals	0
7,8,9	Rv2878c	*mpt53*	A22T	Soluble secreted antigen Mpt53 precursor	cell wall and cell processes	0
7,8,9	Rv2951c	-	V310A	Possible oxidoreductase	intermediary metabolism and respiration	0
7,8,9	Rv3240c	*secA1*	V153G	Probable preprotein translocase SecA1 1 subunit	cell wall and cell processes	0
7,8,9	Rv3299c	*atsB*	G62V	Probable arylsulfatase AtsB (aryl-sulfate sulphohydrolase) (sulfatase)	intermediary metabolism and respiration	0
7,8,9	Rv3455c	*truA*	G74V	Probable tRNA pseudouridine synthase a TruA (pseudouridylate synthase I) (pseudouridine synthase I) (uracil hydrolyase)	information pathways	0
7,8,9	Rv3700c	-	L30V	Conserved hypothetical protein	intermediary metabolism and respiration	0
7,8,9	Rv3741c	-	T160A	Possible oxidoreductase	intermediary metabolism and respiration	0
